# Upconversion Nanocrystal Doped Polymer Fiber Thermometer

**DOI:** 10.3390/s20216048

**Published:** 2020-10-24

**Authors:** Jonas Thiem, Simon Spelthann, Laurie Neumann, Florian Jakobs, Hans-Hermann Johannes, Wolfgang Kowalsky, Dietmar Kracht, Joerg Neumann, Axel Ruehl, Detlev Ristau

**Affiliations:** 1 Institut of Quantum Optics, Leibniz University Hannover, Welfengarten 1, D-30167 Hannover, Germany; j.thiem@lzh.de (J.T.); s.spelthann@lzh.de (S.S.); d.ristau@lzh.de (D.R.); 2 Laser Zentrum Hannover e.V., Hollerithallee 8, D-30419 Hannover, Germany; d.kracht@lzh.de (D.K.); j.neumann@lzh.de (J.N.); 3 TU Braunschweig, Institut für Hochfrequenztechnik, Schleinitzstaße 22, D-38106 Braunschweig, Germany; laurie.neumann@ihf.tu-bs.de (L.N.); florian.jakobs@ihf.tu-bs.de (F.J.); h2.johannes@ihf.tu-bs.de (H.-H.J.); wolfgang.kowalsky@ihf.tu-bs.de (W.K.); 4 Academic Alliance Braunschweig-Hannover QUANOMET, 30167 Hannover, Germany; 5 Cluster of Excellence PhoenixD, 30167 Hannover, Germany

**Keywords:** upconversion nanocrystals, polymer fiber, optical thermometer

## Abstract

In recent years, lanthanide-doped nanothermometers have been mainly used in thin films or dispersed in organic solvents. However, both approaches have disadvantages such as the short interaction lengths of the active material with the pump beam or complicated handling, which can directly affect the achievable temperature resolution. We investigated the usability of a polymer fiber doped with upconversion nanocrystals as a thermometer. The fiber was excited with a wavelength stabilized diode laser at a wavelength of 976 nm. Emission spectra were recorded in a temperature range from 10 to 35 ${}^{\circ }$C and the thermal emission changes were measured. Additionally, the pump power was varied to study the effect of self-induced heating on the thermometer specifications. Our fiber sensor shows a maximal thermal sensitivity of 1.45%/K and the minimal thermal resolution is below 20 mK. These results demonstrate that polymer fibers doped with nanocrystals constitute an attractive alternative to conventional fluorescence thermometers, as they add a long pump interaction length while also being insensitive to strong electrical fields or inert to bio-chemical environments.

## 1. Introduction

Temperature sensing is a part of daily life, and progress in temperature sensor development continues to go hand in hand with the growing demands from various application fields. Besides classical measurements like body temperature, there are various areas of usage under harsh environmental conditions such as radio frequency impulses or electromagnetic impulses. With the continuous progress in many research fields, new challenges for temperature measurement arise, e.g., in vivo temperature measurement combined with magnetic resonance imaging [1] or temperature monitoring of batteries for electromobility [2]. Another challenging field is monitoring in corrosive conditions that impose requirements for sensors and demand creative solutions [3].

Many currently used sensors are simple and cost-effective but exhibit weaknesses depending on the measurement principle and used materials. Temperature sensing is mostly addressed via electrical sensors, like the PT100 or PT1000, which rely on thermally induced resistance changes. However, they are highly sensitive to electrical fields and prone to corrosive environments.

Recently, optically excited lanthanide-doped nanocrystals (NCs) have been established for optical temperature sensing due to their temperature-dependent emission characteristics. The measurement principle is based on the fact that neighbouring energy levels of trivalent lanthanide ions are thermally coupled, so the occupation obeys the Boltzmann distribution. The fluorescence intensity ratio (FIR) from thermally coupled energy levels is, thus temperature dependent. This results in a robust measurement as most external effects like fluctuations in pump power affect both emission lines in the same way, without modifying the ratio.

Different classes of NCs have been applied as an optical nanothermometer (NT), as varying the active ions can maximize the thermometer efficiency, e.g., by adapting to the spectral characteristics of different host media. Among these crystal systems are, e.g., Y${}_{3}$Al${}_{5}$O${}_{12}$:Nd${}^{3+}$ (Nd:YAG) [4], LiYF${}_{4}$:Pr${}^{3+}$ (Pr:YLF) [5] and, most commonly, NaYF${}_{4}$:Er${}^{3+}$,Yb${}^{3+}$ (Er,Yb:YNaF) [6]. The thermal sensitivities of these NT spread around 1%/K and the emission wavelength ranges from the visible to the IR spectrum. One difference between the crystal classes is the excitation mechanism, which is highly nonlinear for Er,Yb:YNaF due to the anti-Stokes-like multiphoton upconversion (UC). Typical NT are dispersed in an organic solvent or doped in thin films. While still offering the robust measurement principle, both are disadvantageous, because either the sensor is difficult to handle, as the solvent may evaporate, or the interaction length is very short.

Contrary to this, fiber-based temperature sensors are easy to handle and are less affected by the optical characteristics of the sample, like, e.g., spectral absorption, as the light is only propagating inside the fiber. A well known example is the Bragg gratings, inscribed in silica fibers. These fibers sense temperature via the shift in the grating constant, which affects the transmission or reflection of an optical signal. Another type of passive-fiber-based thermometer relies on thermally induced changes in the refractive index. These sensors can be manufactured from polymer fibers (POF) and reach sensitivities of 0.115%/K [7], while operating in a temperature range between −55 and 70 ${}^{\circ }$C. Despite all existing advantages, these fiber sensors are not always applicable, because they are prone to errors due to mechanical stress such as strain or rotation.

We propose a combination of NTs and fiber temperature sensors by doping Er:Yb:YNaF NCs into a POF. This sensor concept merges the easy handling and increased pump interaction length of fiber sensors with the robustness of fluorescence ratio measurements from NTs. The use of POFs with large cores and high numerical apertures allows for easy coupling and reduces misalignment errors. POFs show good mechanical stability against fractures and high elongation at break, making them a promising host for stable light guiding. NTs based on Er,Yb:YNaF are highly sensitive to temperature changes, exhibiting one of the best sensitivities of NTs with 1.2–1.4%/K. The doping of POFs with this NC is chemically feasible and has recently been demonstrated [8]. Setting aside the fiber design, polymer doped with NT could open up additional application areas in additive manufacturing, as flexible light guiding structures can be manufactured from polymer by 3-D printing [9] and hence be used in integrated photonics devices. This would enable, e.g., multi-functional lab-on-a-chip components for both measuring sample temperatures and monitoring the device operating conditions.

We investigated the temperature-dependent emission characteristics of such a UC-NC doped POF sensor and determined the temperature resolution for statically and dynamically changing signals at low bandwidth. As monitoring battery temperature in the field of electromobility faces growing demand, a plate capacitor was used to show the insensitivity to electrical fields up to 12.5 kV/cm.

## 2. Fluorescence Thermometry

**Figure 1:**
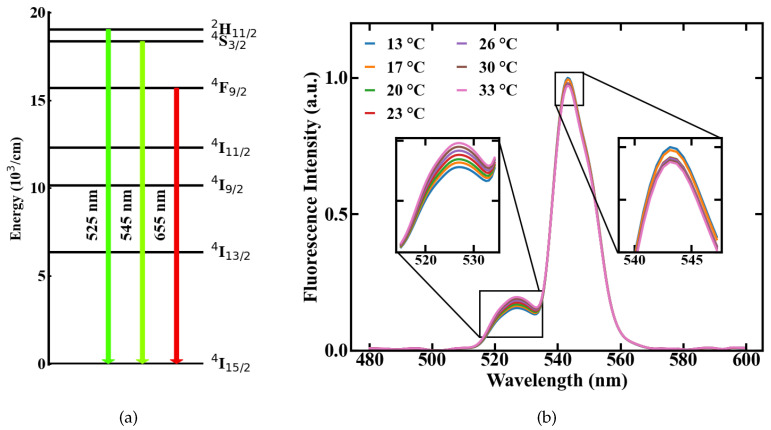
Temperature-dependent emission of a upconversion-nanocrystals (UC-NC)-doped POF, (**a**) involved energy levels of Er${}^{3+}$, (**b**) fluorescence spectra measured at different temperatures.

Optical thermometry utilizes spontaneous emission. The emission intensity of the fluorescence lines of excited materials is determined by the population density of the involved energy states. As described by the Boltzmann equation, the population densities *N*${}_{2}$ and *N*${}_{1}$ of neighbouring energy levels *E*${}_{2}$ and *E*${}_{1}$, in our system ${}^{2}H_{11/2}$ and ${}^{4}S_{3/2}$ in Figure 1a, are thermally coupled. The fluorescence originating from these energy levels is influenced by temperature changes. The correlation between two emission lines can, therefore, be expressed by the FIR [6]

(1) $$FIR=\frac{I_{2}}{I_{1}}\propto \frac{N_{2}}{N_{1}}=A\exp\left(\frac{-\Delta E}{k_{B}T}\right),$$

where *k*${}_{B}$ is the Boltzmann constant, *T* is the temperature, Δ*E* is the energy gap between the two emission levels and *A* is a constant, which accounts for different material specific effects like the degeneracy of the energy states. Following this, a change in temperature results in a shift in the FIR. This effect is shown in Figure 1b on the basis of two emission spectra measured from Er,Yb:YNaF doped in a POF at different temperatures of 13 and 33 ${}^{\circ }$C, respectively. The involved energy level transitions of the Erbium ions are ${}^{2}H_{11/2}$→${}^{4}I_{15/2}$ and ${}^{4}S_{3/2}$→${}^{4}I_{15/2}$ with the corresponding emission centered around 525 and 545 nm, respectively, shown in Figure 1a.

**Figure 2:**
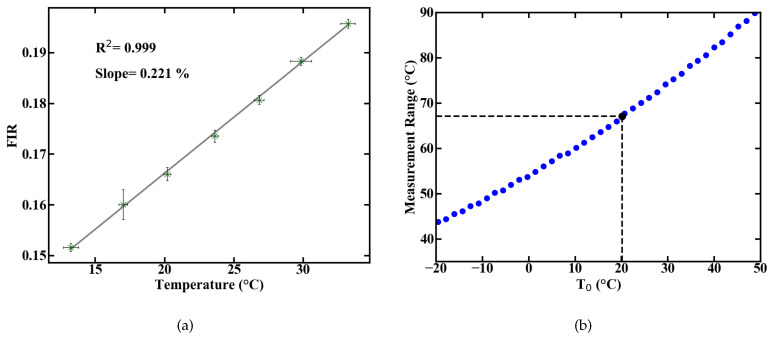
Linear approximation of the measured FIR over temperature with error bars resulting from temperature monitoring via TMP36 sensor and averaging over recorded spectra (**a**), and theoretical prediction of the measurement range (**b**).

Spectral integration of these emission lines and the division of their intensities yields the FIR. The correlation between FIR and temperature is exponential, according to Equation (1). For a simplified usage, the measurement range can be reduced to a temperature range where Equation (1) can be approximated by a linear fit. A representative evolution of the measured FIR with rising temperature, inside such a linear regime with R${}^{2}$ > 0.99, is shown in Figure 2a.

The measurement ranges centered around a temperature value T${}_{0}$ with linear correlation can be calculated from the energy gap ΔE between the levels ${}^{2}H_{11/2}$ an ${}^{4}S_{3/2}$ of Er${}^{3+}$ [10]. The resulting ranges are shown in Figure 2b and the measurement range at room temperature amounts to approximately 67 K below and above the room temperature mark. Aside from the measurement range, the most important parameters of a sensor are its sensitivity and thermal resolution Δ*T*. The relative sensitivity

(2) $$S=\frac{1}{FIR_{0}}\frac{dFIR}{dT}$$

depends on *FIR*${}_{0}$ (meaning the FIR at the central temperature of the measurement range) and the derivative of *FIR* [5]. The minimal thermal resolution Δ*T* can be deduced from Equation (1) by discretising the differential term dFIR/dT and rearranging the equation to

(3) $$\Delta T=S^{-1}FIR_{0}^{-1}\Delta FIR.$$

In terms of practical measurements, this can be understood as the apparent temperature deviation originating from the measurement error of the emission ratios. This error Δ*FIR* can be calculated from the uncertainties of the single ratios, according to the error propagation, as

(4) $$\Delta FIR=|\frac{\partial FIR}{\partial I_{2}}|\Delta I_{2}+\left|\frac{\partial FIR}{\partial I_{1}}\right|\Delta I_{1}=\frac{\Delta I_{2}}{I_{1}}+\frac{I_{2}}{I_{1}^{2}}\Delta I_{1}.$$

To further reduce the measurement error, multiple data acquisitions, e.g., in combination with a moving average, is useful.

## 3. Manufacturing of Upconversion Nanocrystal-Doped POF

**Figure 3:**
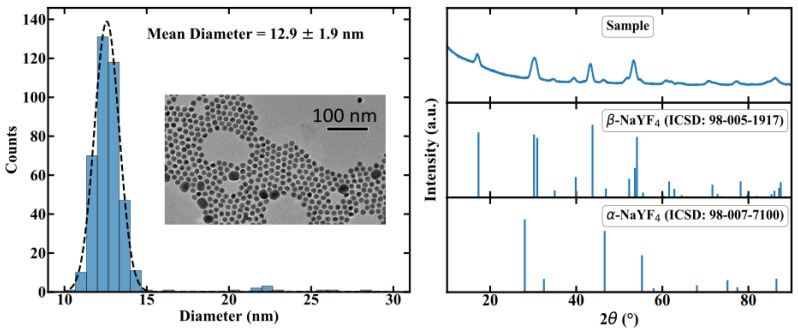
Characterization of the size distribution and the crystal phase of the manufactured NCs by using TEM and XRD, respectivly. Further details can be found in [8].

In this section, we will provide a brief description of the manufacturing process of the NC doped POF. A detailed description can be found in [8]. In order to incorporate the NC in a polymer matrix, an in situ polymerisation process was chosen. While standard POFs are typically made of poly-methyl methacrylate (PMMA) [11], the NC-doped PMMA made from methyl methacrylate solution is blurry, revealing a decrease in transparency. Furthermore, NCs are likely to agglomerate and precipitate in PMMA. Co-polymers consisting of mixed methyl methacrylate (MMA) and cyclohexyl methacrylate (CHMA) enable highly transparent polymers [12] and provide no agglomeration. Therefore poly(CHMA-co-MMA) (PMMA/PCHMA) was chosen as the host material for the fibers. For the active material, we chose Er,Yb:YNaF UC-NC, because of the well-researched parameters in thermometer applications. The luminescence of these NC depends on the crystal phase, specifically the weakly luminescent cubic α-phase and the highly luminescent hexagonal β-phase. Therefore, in addition to Er${}^{3+}$ and Yb${}^{3+}$, Gd${}^{3+}$ ions were doped to the crystal to adjust the phase [13]. The resulting chemical formula of our NC is β-NaYF:Yb(20),Er(2),Gd(20). For the sake of simplicity, and since no important optical transitions occur in Gd${}^{3+}$, we use Er,Yb:YNaF as an abbreviation. The NCs were synthesized using thermal decomposition in an Oswald ripening process. Transmission electron microscopy revealed a size distribution of 12.9 ± 1.9 nm, as shown in Figure 3. Thus optical scattering is assumed to be weak.

The X-ray diffraction measurement shows hexagonal β-phase NaYF${}_{4}$ particles leading to a well defined crystal structure for the emitting lanthanide ions, also depicted in Figure 3. The UC-NCs were surrounded by oleic acid ligands to provide better solubility in the co-monomer.

**Figure 4:**
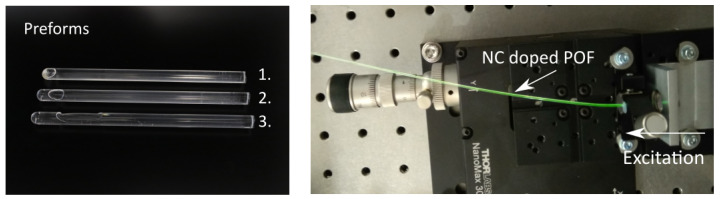
Shown in the left picture are three comonomer polymer preforms 1, 2, and 3 doped with 0.5 wt%, 0.1 wt%, and 0 wt% of UC-NCs, respectively. The doping concentration used in the following experiments was 0.5 wt%. Presented in the right picture is the green emission of an UC-NC doped POF after excitation with 976 nm.

Preforms were prepared using radical polymerization in bulk with 0.03 mol% lauroyl peroxide as an initiator and 0.2 mol% *n*-butyl-mercaptan as a chain transfer agent. Dried UC-NCs were mixed with the initiator and the chain transfer agent in a nitrogen-saturated mixture of co-MMA-CHMA. The solution was polymerized and the final UC-NC:poly-MMA-co-CHMA preforms were drawn in a drawing tower. Fiber core diameters of 980 μm were provided. In a second step, the fiber was coated with acrylate and drawn through a nozzle, leading to fiber diameters of 1000 μm. The cladding was cured with UV light afterwards. Further details on the manufacturing and preparation process for the POFs can be found in [8,14,15,16]. An exemplary illustration of different comonomer polymer preforms as well as emission from a doped fiber excited with 976 nm is shown in Figure 4.

## 4. Measurement Setup

**Figure 5:**
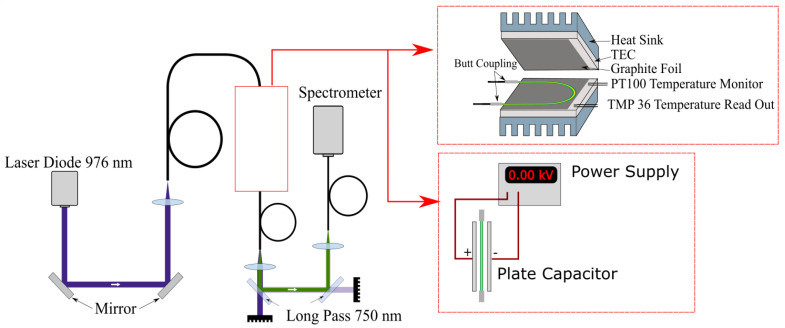
Setup used to characterize the sensor performance. Measurements of the thermal influence of fluorescence emission were performed using thermoelectric cooling elements (TEC), as shown in the upper inset. To identify the effects of electrical fields on the sensor the TEC were substituted with a plate capacitor, depicted in the lower inset.

**Figure 6:**
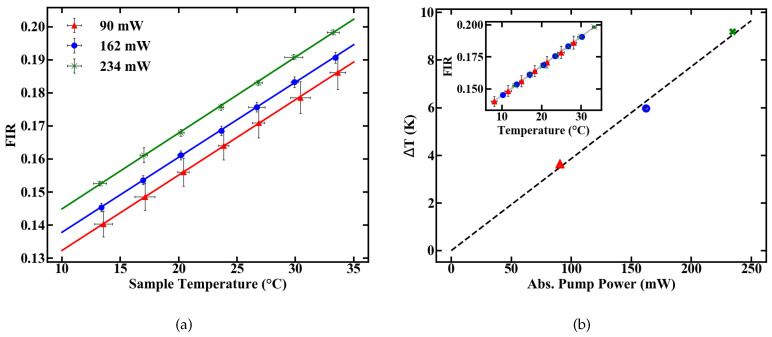
Temperature-induced changes in the FIR at different pump powers (**a**) and an additional temperature shift accounting for pump induced heating (**b**).

We developed a setup to characterize the fiber thermometer, as depicted in Figure 5. A wavelength-stabilized laser diode (H21416313, Oclaro, San Jose, CA, USA) (Oclaro is now part of Lumentum and the specific type of laser diode may not be available for purchase, but any laser diode with an emission wavelength of 976 nm and an output power of a few Watt should also be sufficient to perform the experiments.) with an maximum output power of 20 W, an central output wavelength of 976 nm and a spectral bandwidth of approximately 1 nm was used to excite emission from the UC-NC-doped POF. Fluctuations in the pump power were measured to be below 1–2% depending on laser power and are neglected in the following. This is feasible because they do not affect FIR measurements, as explained before, and fiber temperature as shown in Figure 6. Emission spectra were recorded using a fiber coupled USB-spectrometer (AvaSpec-ULS2048x64-EVO, Avantes, 7333 NS Apeldoorn, The Netherlands, and Black-Comet-SR, Stellar Net Inc., 14390 Carlson Circle, Tampa, FL, USA) with an integration time of 1 s. The beam was delivered by coupled commercial fused silica fibers, with matching numerical apertures of 0.5 and diameters of 400 μm to reduce coupling errors between the fibers. A real time fitting routine was applied to assign the measured spectra to the corresponding energy levels. Because different line-broadening mechanisms are expected, due to varying emission rates from the lanthanide ions depending on their position in the NC [17] and the amorphous host polymer, Voigt profiles were used as fitting functions.

To characterize the thermal response of the sensor, the POF was contacted to thermoelectric cooling elements (TEC) using graphite foil. The temperature for the control loop was monitored with a PT100, and additionally the temperature was recorded in real time using a electronic sensor (TMP36) connected to a power supply and an A/D converter. The accessible temperature range was 10 to 35 ${}^{\circ }$C, and actual application is limited in its temperature range by anything other than material properties of the co-polymer. Additional measurements were performed to investigate the influence of electric fields on the sensor. For this reason, the setup was modified by exchanging the TECs with a plate capacitor, as shown in Figure 5. The maximal applied electric field was 12.5 kV/cm. Fluorescence spectra were recorded for different field strengths with a spectrometer resolution of 0.5 nm.

## 5. Fluorescence Intensity Ratio Measurements and Sensor Calibration

Three pump power levels were chosen to characterize the thermometer specifications of the fiber. To calculate the deposited pump power, a cut back measurement was performed and the pump absorption rate of the doped POF was determined to be 6.7 dB/m. The FIR for different temperatures was then determined at levels of absorbed pump power inside the fiber of 90, 162 and 234 mW, respectively. The results are given in Figure 6a.

The pump-induced temperature change Δ*T* inside the fiber can be estimated by the offset between the calibration lines in Figure 6a. Assuming 0 K heating at 0 mW of absorbed pump power, the resulting temperatures, as given in Figure 6b, are derived from a linear fit. These results reveal a heating of 10 K relative to the sample temperature at an absorbed pump power of 234 mW. The longitudinal temperature distribution inside the fiber is assumed to be homogeneous, which is justified for either weakly doped or short fibers, because, for these fibers, absorption can be linearly approximated. For example, at an absorption rate of 6.7 dB/m, approximately 2.5% of pump power is absorbed per centimeter propagation inside the fiber. This means only minor variations in the amount of absorbed pump power between, e.g., the first length increment of the fiber and the last, which, combined with heat flux inside the fiber, justify the assumption.

Nevertheless, the increase in temperature has to be considered, especially for biomedical applications. Since the fluorescence output power of the fiber is negligible compared to the absorbed pump light, and scattering can be assumed to be weak at 976 nm, most of the absorbed pump power is converted into heat. The absorption values can, therefore, be used as a figure to estimate the heat deposition to the surrounding media. Because of this, the value of absorbed pump power is more important for the specifications of the thermometer than the amount of incident pump power. Consequently, all the following power values, if not otherwise stated, correspond to the absorbed power inside the fiber sensor with a length of 15 cm and a diameter of 1 mm. This length was chosen due to practical reasons. In general, the length of the fiber should only influence the sensor performance in extreme cases for short and long fibers, as the fluorescence emission tends to saturate in the absence of stimulated emission after a short fiber length [18]. The coupling efficiency from the pump light delivery fiber to the POF was roughly 85%.

**Figure 7:**
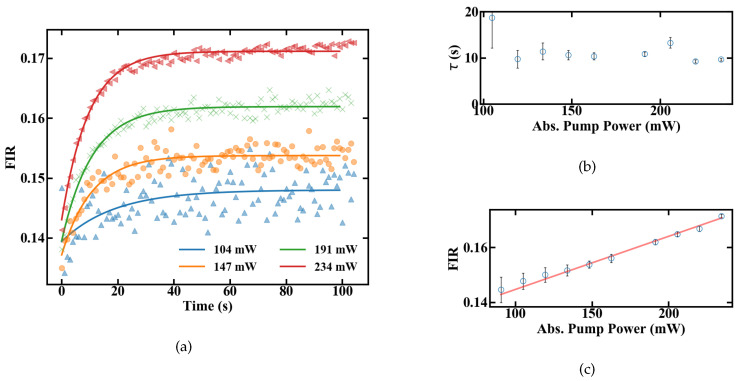
FIR of a 15-cm-long doped POF at different absorbed pump powers shown in (**a**) changes after the pump laser is turned on (**b**) time until equilibrium is reached (**c**) equilibrium levels.

The slope dFIR/dT is calculated by a linear regression with a fit quality of R${}^{2}$ > 0.99. Figure 6 shows a similar slope for all pump powers of around 0.22%. In contrast to the slope, the absolute FIR at 22 ${}^{\circ }$C nominal sample temperature varies for different pump powers. The rise in the emission ratio with increased pump power illustrates the influence of self-induced heating on the thermometer, depicted in Figure 6b. A calibration in terms of induced temperature change inside the fiber and its effect on the FIR is therefore necessary. This was carried out by measuring the variation in the FIR without externally controlling the heat of the sample. The results over time for different pump powers are shown in Figure 7. Figure 7a depicts the rise in the FIR over time. Since these measurements were performed at room temperature, the corresponding FIR can be estimated from the first acquisition points to approximately 0.130–0.135. Starting from this, thermal equilibrium inside the fiber is reached after roughly 60 s for all absorbed pump powers studied, as depicted in Figure 7a. Fitting exponential functions to the data yield the time until the 1/e point is reached. This duration is given in Figure 7b, with values around 10 s.

To estimate heating of the sample, the FIR values with and without heat control can be compared. At 234 mW of absorbed pump power, the FIR in Figure 6a at 21 ${}^{\circ }$C nominal sample temperature and the FIR reached in thermal equilibrium in Figure 7c both have a value of approximately 0.17. This indicates that they were also measured at matching sample temperatures. Since the measurements for the unregulated sample were carried out at a room temperature of around 20–20.5 ${}^{\circ }$C, heating of the sample, not of the fiber itself, is limited to the range of 0.5–1 K, even for the maximal measured amount of absorbed pump power, and should be even smaller for lower absorbed power levels. Therefore, a significant influence of the fiber temperature on the sample for the investigated pump powers seems to be unlikely, and heat dissipation is fast enough. Alternatively, if the sample temperature would increase by, e.g., 10 ${}^{\circ }$C the thermal equilibrium FIR measured without heat control, Figure 7c, would be significantly larger, corresponding to a controlled sample temperature at, e.g., 30 ${}^{\circ }$C in Figure 6a, with an FIR of 0.19. The difference between sample temperature and fiber temperature seems to be constant, because the slope dFIR/dT is the same for different pump powers. Concerning the measurement accuracy, sample heating would therefore only create an offset for the temperature read-out, which can be calibrated for different samples with different heat dissipation rates. In this work, where sample heating is small, especially for low pump powers, and heat dissipation inside the sample is fast enough, this offset is neglected and the focus is placed on the accuracy, with which temperature changes can be measured.

**Figure 8:**
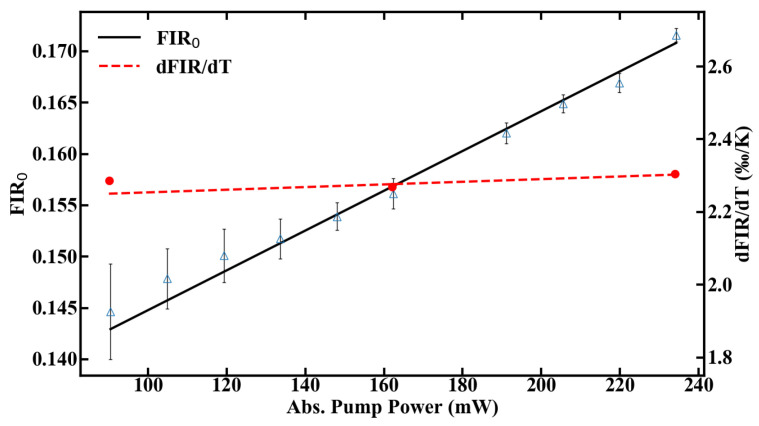
Calibration range of the POF thermometer.

Besides heating effects, the right choice of pump power has additional consequences in terms of temperature resolution, as the signal-to-noise ratio scales with the amount of fluorescence emission. Therefore, compromises need to be made between accuracy of the measurement and tolerable heat input to the system. The thermal sensitivity of the sensor, defined in Equation (2), can be calibrated for different pump powers by using the FIR${}_{0}$ given in Figure 8 and the slope dFIR/dT from Figure 6a. The range, in which the sensor can be calibrated, is shown in Figure 8.

## 6. Sensitivity and Temperature Resolution

**Figure 9:**
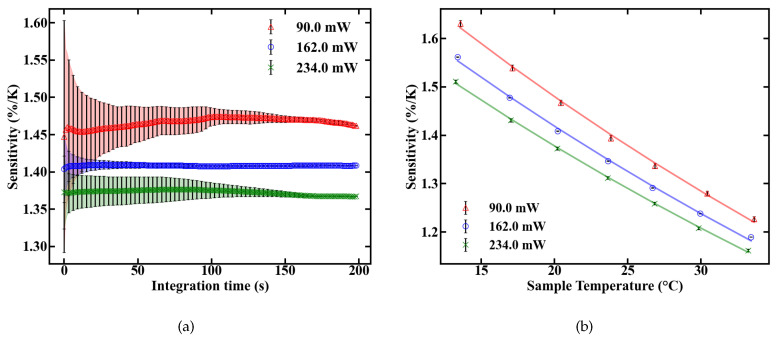
Sensitivity of the UC-NC-doped fiber sensor as a function of integration time (**a**) and temperature (**b**).

The sensitivity of the fiber sensor, shown in Figure 9, was calculated from the values of FIR and dFIR/dT. The maximum sensitivity of the POF sensor at room temperature is 1.45%/K at 90 mW of absorbed pump power. The sensitivity decreases with rising temperature inside the fiber, as expected, which is depicted in Figure 9b.

**Figure 10:**
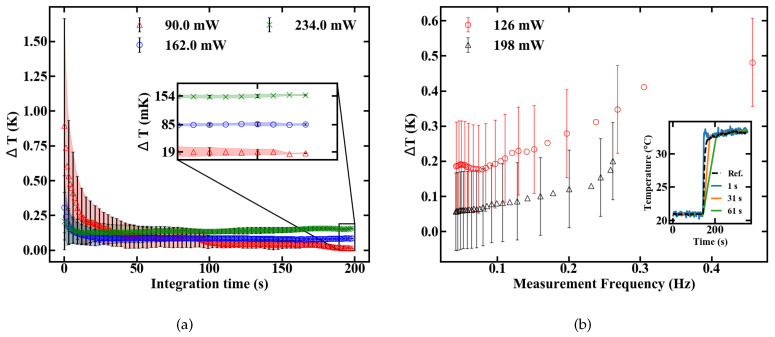
Dependence of the temperature resolution Δ*T* from the integration time (**a**) and measurement frequency as measured by a step response (**b**).

The temperature resolution depends, besides the sensitivity value, on the signal to noise ratio (SNR) of the measurement setup. The minimum resolutions at different pump powers are shown in Figure 10a to be 154, 85 and 19 mK for 234, 162 and 90 mW, respectively.

Table 1 gives a comparison of these sensor characteristics of the doped POF sensor and both a passive POF thermometer and Er,Yb:YNaF NTs.

In terms of temperature resolution, additional compromises can be made concerning pump power and integration time. Low pump power ensures a minimum resolution only at long integration times because of a reduced SNR. They should, therefore, be used for measurements of slowly changing temperatures. On the contrary, high pump power maximizes the SNR, but reduces the sensitivity while also increasing the heat transferred to the sample. They are, therefore, preferable for fast thermal changes inside robust and thermally highly conductive samples. A step response was measured to obtain a better understanding of the dynamic temperature resolution. A sudden temperature change was applied and the sensor output was analysed by applying moving averages with a varying sampling duration, as shown in the inset of Figure 10b. The temperature resolution in Figure 10b is calculated by the standard deviation from the reference signal, which was measured with an electrical sensor. The measurement frequency is defined as the inverse settling time of the sensor. The results indicate that oscillating temperatures can be measured with a certainty of roughly 0.5 K, if the frequency does not excel 0.4 Hz, as depicted in Figure 10b.

## 7. Influence of Electric Fields

**Figure 11:**
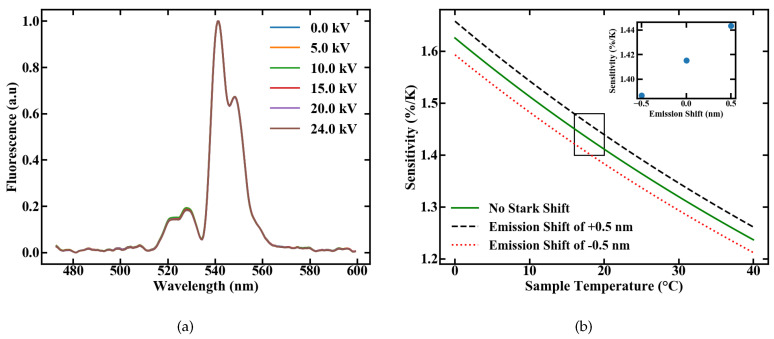
(**a**) Emission spectra of a Er,Yb:YNaF POF inside a plate capacitor for different applied fields and (**b**) variation in the sensitivity caused by an assumed shift in the emission lines of 0.5 nm.

We investigated the influence of electric fields on the sensor performance by measuring the emission spectra, after excitation with 1.2 W of pump power, at various voltages applied to the plate capacitor ranging from 0 to 24 kV; the corresponding setup is shown in Figure 5. The plates were set to a distance of 2 cm, so that the applied field ranges from 0 to 12.5 kV/cm with multiple discharges at the latter value. The normalized emission spectra are shown in Figure 11a. Influences of the electric field on the FIR measurement, like the Stark Effect, would be indicated by emerging peaks or changes in the spectral shape, but none of these effects are visible with the used spectrometer resolution.

To estimate the maximal possible error caused by an emission shift of 0.5 nm, which corresponds with our spectrometer resolution, we calculated the sensitivity, according to [5] with

(5) $$S=\frac{\Delta E}{k_{B}T^{2}}$$

for different energy gaps. The assumed value for the energy gap is therefore the energy gap between the levels ${}^{2}H_{11/2}$ and ${}^{4}S_{3/2}$ of Er${}^{3+}$ [10], with an applied shift corresponding to the measurement uncertainty of 0.5 nm. The results are shown in Figure 11b, with a sensitivity ranging from 1.38 to 1.44%/K. If only the linear Stark Effect as the perturbation source is considered, the maximum deviation in sensitivity through external electric fields can be estimated to a value of 50 ppm/(kV/cm). Although a complete shift in one emission band by 0.5 nm can not be observed, this calculation again highlights that high sensitivities could still be reached, even inside strong electric fields. The fiber sensor therefore works independently of external electric fields in the measured range.

## 8. Conclusions

We showed that by doping upconversion NCs inside POFs, temperature sensors with an excellent sensitivity of 1.45%/K and resolution of 19 mK can be achieved. The associated values are given in Table 1. Besides the higher sensitivity, our sensor shows additional advantages compared to other passive fiber sensor concepts, like the one presented in [7]. As previously mentioned, the measurement of FIR is insensitive to fluctuations in the pump power, therefore eliminating the need for compensation techniques. Since passive sensors often rely on the difference in transmittance between two wavelengths, they also need at least two light sources. By applying NTs, we reduced this to only one laser diode, which is needed to excite emission, thus further simplifying the usage of the sensor for applications and potentially reducing the cost of a measurement setup.

We characterized the system within a temperature range of 13 to 33 ${}^{\circ }$C, but there is no indication that the sensor is limited to these values. The measurement range solely depends on the integrity of the host material. A POF sensor has been successfully demonstrated between −55 to 70 ${}^{\circ }$C [7]. The operating range of our temperature sensor therefore matches the required temperature range of 15 to 50 ${}^{\circ }$C [2] for monitoring lithium batteries. To investigate whether the sensor could be used for this application, we measured the influence of electrical fields and we found no influence on the performance for electric fields up to 12 kV/cm.

## 9. Outlook

Since the polymer host material also shows good bio compatibility, our sensor constitutes an ideal candidate to monitor temperatures in various medical applications. Further simplification of the setup could be realized by adding reflecting films to the fiber end facets similar to the approach in [24]. This preparation would allow a more compact setup and would be especially beneficial for endoscopic temperature monitoring. To increase the spatial resolution, the doped fiber containing the NCs could be spliced to undoped fiber sections. Combining these benefits opens up endoscopic temperature measurements, e.g., inside magnetic resonance imaging, although it should be mentioned that the pump-light-induced heating might be a drawback for such medical applications. A possible solution to this could be the usage of smaller cores combined with improvements in emission measurement. By reducing the core size, the same pump energy density and the same population density could be reached at reduced absolute values of pump power. This would minimize the absolute value of deposited heat but also reduce the absolute emission output, and therefore, potentially, accuracy. Further research should also focus on the utilisation of different polymers to the reduce absorption and, therefore, heating of the host material.

The application areas of our NT-polymer combination can also be significantly broadened by setting aside the fiber design, as much more flexible light-guiding structures can be manufactured from polymer by 3-D printing [9]. These techniques enable the integration of the NT into more complex photonic structures such as multi-functional lab-on-a-chip components.

## Figures and Tables

**Table 1:** Comparison of different temperature sensors.

	UC-POF	Er,Yb:YNaF NTs [6,19,20,21]	POF Sensors [7,22,23]
Sensitivity (%/K)	1.45	1.2–1.1	0.192–0.104
Temperature Resolution (mK)	19	83	–
Temperature Range (${}^{\circ }$C)		−50–210	−55–70
